# Intrathyroidal Parathyroid Carcinoma: Report of an Unusual Case and Review of the Literature

**DOI:** 10.1155/2013/198643

**Published:** 2013-07-14

**Authors:** Lizette Vila Duckworth, William E. Winter, Mikhail Vaysberg, César A. Moran, Samer Z. Al-Quran

**Affiliations:** ^1^Department of Pathology, University of Florida College of Medicine, P.O. Box 100275, Gainesville, FL 32610-0275, USA; ^2^Department of Otolaryngology, University of Florida College of Medicine, Gainesville, FL 32610-0264, USA; ^3^Department of Pathology, MD Anderson Cancer Center, Houston, TX 77030, USA

## Abstract

Intrathyroidal parathyroid carcinoma is an exceedingly rare cause of primary hyperparathyroidism. A 51-year-old African American female presented with goiter, hyperparathyroidism, and symptomatic hypercalcemia. Sestamibi scan revealed diffuse activity within an enlarged thyroid gland with uptake in the right thyroid lobe suggestive of hyperfunctioning parathyroid tissue. The patient underwent thyroidectomy and parathyroidectomy. At exploration, a 2.0 cm nodule in the usual location of the right inferior parathyroid was sent for intraoperative frozen consultation, which revealed only ectopic thyroid tissue. No parathyroid glands were identified grossly on the external aspect of the thyroid. Interestingly, postoperative parathyroid hormone levels normalized after removal of the thyroid gland. Examination of the thyroidectomy specimen revealed a 1.4 cm parathyroid nodule located within the parenchyma of the right superior thyroid, with capsular and vascular invasion and local infiltration into surrounding thyroid tissue. We present only the eighth reported case of intrathyroidal parathyroid carcinoma and review the literature.

## 1. Introduction

Parathyroid carcinoma is a rare clinical entity comprising 0.5 to 2% of patients who present with primary hyperparathyroidism. Equally unusual is the presence of an intrathyroidal parathyroid gland (0.2%), which originates from aberrant migration of the parathyroid gland(s) from the third and fourth branchial pouches [1]. We report an unusual case of parathyroid carcinoma arising from intrathyroidal parathyroid tissue and review the related literature.

## 2. Case Report

**Figure 1:**
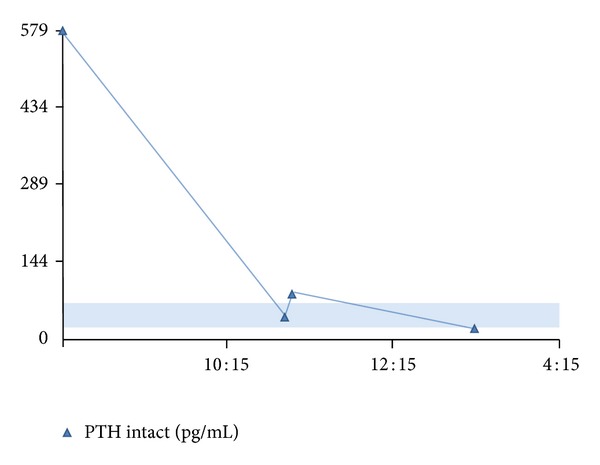
Intraoperative monitoring of PTH levels. Graph depicts marked decrease in parathyroid hormone levels following thyroidectomy. PTH hormone dropped initially from 579 to 40 pg/mL, then 83 pg/mL, and finally to 16 pg/mL. (PTH level is on *y*-axis, time is on *x*-axis, and shaded box represents PTH reference range from 15 to 65 pg/mL.)

A 51-year-old African American female presented to our institution with thyromegaly and fatigue. Thyroid function tests were consistent with hypothyroidism. Thyroid ultrasound and radioactive uptake scan demonstrated diffuse goiter, with a dominant cold nodule (4.0 × 2.6 × 2.5 cm) in the inferior right thyroid lobe. The patient elected not to undergo biopsy and/or thyroidectomy at that time. A repeat radioactive uptake scan three years later again demonstrated diffuse goiter with a stable cold nodule in the right inferior thyroid lobe. Fine needle aspiration of this nodule was performed which yielded scant colloid and follicular cells in an occasional microfollicular pattern, indeterminate for a follicular neoplasm. Additionally, the patient was found to have elevated calcium levels in the range from 12.6 to 14.0 mg/dL (reference range 8.0–10.6 mg/dL), low phosphorous levels from 2.4 to 2.6 mg/dL (2.7–4.5 mg/dL), and markedly elevated intact parathyroid hormone (PTH) ranging from 303 to 579 pg/mL (15–65 pg/mL). In retrospect, the patient had elevated calcium levels for several years and had symptoms relating to hypercalcemia including fatigue, bone pain, abdominal pain, and memory loss. Vitamin D levels showed a low 25-hydroxyvitamin D of 7 ng/dL (10–50 ng/mL) and high 1,25 dihydroxyvitamin D of 103 pg/mL (15–60 pg/mL), consistent with primary hyperparathyroidism. Sestamibi scan revealed diffuse activity within an enlarged thyroid gland with uptake in the right lobe suggestive of hyperfunctioning parathyroid tissue. Although imaging was not entirely classic for a parathyroid adenoma, this could not be excluded. The patient elected to proceed with thyroidectomy and parathyroidectomy due to compressive symptoms from her goiter and symptomatic hypercalcemia. During neck exploration, a 2.0 cm nodule in the usual location of the right inferior parathyroid, thought to be an adenoma, was removed and sent for intraoperative frozen consultation. Frozen section examination revealed only ectopic thyroid tissue with no parathyroid tissue identified. No additional parathyroid tissue was identified grossly on the external aspect of the thyroid. Interestingly, following removal of the entire thyroid gland, intraoperative PTH levels decreased to 40, then 83, and finally to 16 pg/mL ten minutes postoperatively (see Figure 1).

**Figure 2:**
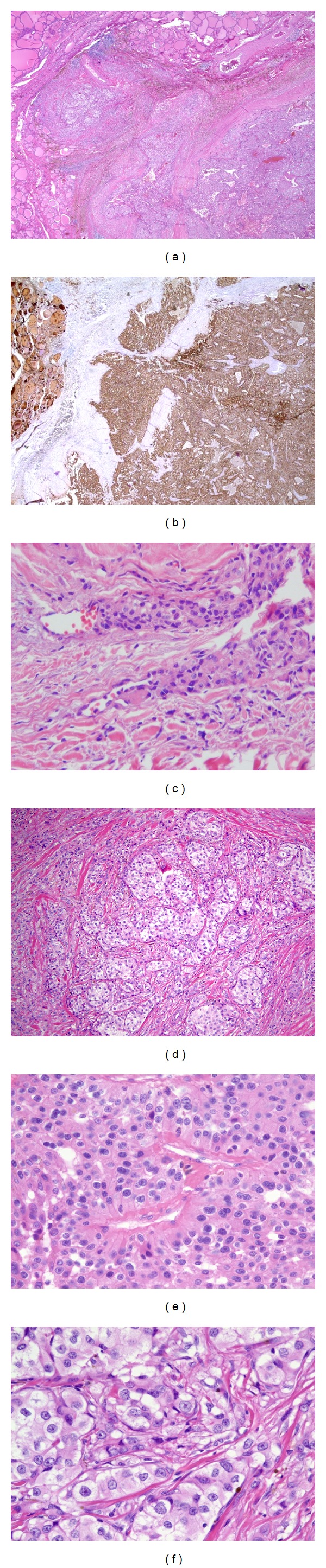
Microscopic appearance of intrathyroidal parathyroid carcinoma. (a) Extracapsular extension (“mushroom projection”) of neoplastic cells through the capsule into surrounding thyroid parenchyma (hematoxylin and eosin stain (H&E); magnification 20x). (b) Immunohistochemistry for parathyroid hormone (PTH) shows strong and diffuse immunoreactivity in tumor cells and highlights areas of capsular invasion. There is nonspecific staining of colloid in thyroid follicles in the upper left (20x). (c) Foci of vascular invasion seen at the periphery of the tumor (H&E; 100x). (d) Trabecular arrangement of tumor cells traversed by thick bands of collagen (H&E; 40x). (e) Focal perivascular arrangement of tumor cells around vessels (H&E; 200x). (f) Cytologic features of tumor cells demonstrating tumor cell monotony with clear to eosinophilic cytoplasm and prominent macronucleoli (H&E; 400x).

Gross examination of the thyroid revealed a diffusely enlarged gland (95.5 g), distorted by multiple soft, red-tan nodules varying from 0.4 to 4.5 cm in size, with a dominant nodule in the right inferior thyroid which had been previously needled. Histologically, these nodules were consistent with adenomatoid nodules (nodular goiter) with focal nonspecific lymphocytic thyroiditis. Cut surface of the thyroid gland revealed an additional 1.4 × 1.4 × 0.9 cm soft, yellow-tan nodule located within the parenchyma of the right superior thyroid. Microscopic examination of this nodule revealed cellular nests of clear to eosinophilic epithelioid cells divided by dense bands of fibrosis and surrounded by a thick fibrous capsule (Figure 2). Areas of invasion through the capsule into surrounding normal thyroid tissue, as well as areas of vascular invasion, were identified at the periphery of the tumor. Focally, the tumor cells had a perivascular arrangement with basally located nuclei. The tumor cells contained hyperchromatic to vesicular nuclei with prominent macronucleoli and occasional intranuclear inclusions. Mitotic count was 3 per 50 high-power fields. Immunohistochemical studies revealed that tumor cells were strongly immunoreactive for PTH and negative for calcitonin, TTF-1, and thyroglobulin, thus confirming that the tumor cells were parathyroid in origin. The tumor cells were also positive for renal cell carcinoma marker (RCC), but negative for CD10, S-100, synaptophysin, chromogranin, and Hep-Par-1. Ki-67 demonstrated a proliferation index of approximately 5% in tumor cells. Two-level VI lymph nodes submitted for evaluation were negative for metastatic carcinoma. Based on the findings of capsular and vascular invasions and local infiltration into the thyroid gland, a diagnosis of parathyroid carcinoma was made. The patient was managed with surgery alone, without adjuvant radiation, and continues to do well with no evidence of recurrent disease after 2.5 years of followup.

## 3. Discussion

Parathyroid carcinoma is an exceedingly rare clinical entity, which occurs equally in males and females with a median age of 45 years. Parathyroid carcinoma often presents as a mass that is adherent to adjacent structures. Laboratory criteria to distinguish parathyroid adenoma from carcinoma are nonspecific. The clinical criteria of local invasion and/or metastases are usually required for the diagnosis of parathyroid carcinoma, as histologic features alone cannot entirely distinguish between an adenoma and carcinoma. Capsular, vascular, and perineural invasions are usually reliable indicators of malignancy. Other histologic features more commonly seen in carcinoma include a trabecular growth pattern with dense fibrous bands, tumor cell necrosis, tumor cell monotony, increased nuclear to cytoplasmic ratio, spindling of tumor cells, prominent irregular macronucleoli, and increased or atypical mitotic figures. In addition, higher expression of Ki-67, lower expression of p27kip1, and cyclin D1 overexpression have been demonstrated in carcinomas versus adenoma/hyperplasia [2–4]. Loss of nuclear parafibromin, a protein encoded by the putative tumor suppressor gene *HRPT2*, has also been shown to be a highly sensitive and specific marker for the diagnosis of parathyroid carcinoma [5].

Approximately 700 cases of parathyroid carcinoma have been reported to date, and, to the best of our knowledge, only seven cases of intrathyroidal parathyroid carcinoma have been previously documented (see Table 1) [6–13]. Ernst et al. reported the first case of intrathyroidal parathyroid carcinoma in a patient presenting with hyperparathyroidism [6]. Crescenzo et al. later presented a case of intrathyroidal parathyroid carcinoma in a patient who presented with a left neck mass [7]. Other presentations of intrathyroidal parathyroid carcinoma have included goiter and symptoms relating to hypercalcemia [9–13]. Herrera-Hernández et al. described the first case of intrathyroidal parathyroid carcinoma occurring in a pediatric patient [12]. Sestamibi scan was helpful in three cases in localizing the parathyroid gland before surgical exploration [8, 10, 12]. Findings of lymphocytic thyroiditis, similar to our case, and a microscopic focus of papillary thyroid carcinoma were detected in a case by Schmidt et al. [9]. Ordoñez et al. described a rare case of intrathyroidal parathyroid tumor producing amyloid, mimicking medullary carcinoma of the thyroid gland [14]. Nonfunctioning intrathyroidal parathyroid carcinomas have not been described.

Recognition of this rare entity is important as parathyroid carcinoma has a high probability of local recurrence and the potential to metastasize to regional nodes and distant sites late in its course. Reported five-year survival rates range from 50 to 85.5%. The presence of macronucleoli, greater than 5 mitoses per 50 high-power fields, and necrosis are regarded by some to be poor prognostic factors associated with recurrent disease [15]. Complete surgical excision is the primary treatment with or without postoperative radiation. Important to exclude in the differential diagnosis are intrathyroidal parathyroid adenoma, follicular thyroid carcinoma (TTF-1 and thyroglobulin positive, PTH negative), and medullary thyroid carcinoma (TTF-1 and calcitonin positive, thyroglobulin and PTH negative). In addition, expression of RCC should not be mistaken for metastatic renal cell carcinoma, as RCC positivity has been demonstrated in several nonrenal tumors, including parathyroid carcinomas [16]. 

## Figures and Tables

**Table 1:** Literature review of published intrathyroidal parathyroid carcinoma cases.

Reference	Case no.	Age/sex	Presentation	Tumor location and size	Treatment	Outcome
Ernst et al., 1993 [6]	1	52/F	Hyperparathyroidism/hypercalcemia	Left thyroid, 2.5 cm	Left thyroidectomy	NED, 4 mo
Crescenzo et al., 1998 [7]	2	60/F	Left neck mass, hyperparathyroidism/ hypercalcemia	Left thyroid, 1.5 cm	Left thyroidectomy, isthmusectomy	NED, 18 mo
Kirstein and Ghosh, 2001 [8]	3	74/M	Hyperparathyroidism	Left thyroid, NR	Left thyroidectomy	NR
Schmidt et al., 2002 [9]	4	76/F	Hyperparathyroidism/hypercalcemia	Right superior thyroid, 3.2 cm	Right thyroidectomy, isthmusectomy	NED, 1 yr
Hussein et al., 2006 [10]	5*	63/F	Hyperparathyroidism/hypercalcemia	Left thyroid, 6.0 cm	Left thyroidectomy	NED, >1 mo
Foppiani et al., 2007 [11]	6	67/F	Hyperparathyroidism/hypercalcemia, multinodular goiter	Right inferior thyroid, 3.0 cm	Total thyroidectomy	NED, 5 yr
Herrera-Hernández et al., 2011 [12]	7	14/F	Hyperparathyroidism/hypercalcemia	Right thyroid, 2.5 cm	Right thyroidectomy	NED, 18 mo
Vila Duckworth et al., present case	8	51/F	Hyperparathyroidism/hypercalcemia, thyromegaly	Right superior thyroid, 1.4 cm	Total thyroidectomy	NED, 2.5 yr

NR indicates not reported; NED: no evidence of disease; mo: months; yr: years.

*Case apparently also described by Temmim et al. 2008 [13].

## Abbreviations

PTH: Parathyroid hormone.
